# Der Schmerz und seine Grenze: Eine phänomenologische Betrachtung chronischer Schmerzen

**DOI:** 10.1007/s00482-023-00776-9

**Published:** 2024-01-09

**Authors:** Judith N. Wagner

**Affiliations:** 1 Klinik für Neurologie, Evangelisches Klinikum Gelsenkirchen, Akademisches Lehrkrankenhaus der Universität Duisburg-Essen, Munckelstraße 27, 45879 Gelsenkirchen, Deutschland; 2 Faculty of Humanities, Department of Philosophy, Charles University, Prag, Tschechien

**Keywords:** Transparenz, Leib, Enaktivismus, Phänomenologie, Merleau-Ponty, Transparency, Lived body, Enactivism, Phenomenology, Merleau-Ponty

## Abstract

Chronische Schmerzen stellen weltweit ein signifikantes sozialmedizinisches Problem mit hohen Folgekosten dar. Als eigenständige Erkrankung werden Schmerzsyndrome jedoch erst seit etwa Mitte des 20. Jahrhunderts angesehen. Schon in der Definition der International Association for the Study of Pain wird deutlich, dass Schmerz ein komplexes, kontextabhängiges, damit aber auch modifizierbares Phänomen darstellt. Die philosophische Herangehensweise an den Schmerz ist mindestens ebenso facettenreich und kann unter den verschiedensten Gesichtspunkten erfolgen. Im Folgenden soll eine Charakterisierung des Schmerzes unter Einbeziehung einer philosophischen – phänomenologisch und enaktivistisch geprägten – Perspektive angestrebt werden. Als Leitstruktur dieser Betrachtung soll das Konzept der leiblichen Grenze dienen: In welcher Beziehung stehen Schmerz und die Wahrnehmung der Leibesgrenzen zueinander? Handelt es sich um eine wechselseitige Einflussnahme? Und ist diese Wahrnehmung zugunsten des Schmerzpatienten modifizierbar? Anhand dieser Überlegungen wird auch deutlich werden, dass die beiden Wissenschaften – Neurowissenschaften und Philosophie – mitnichten Konkurrentinnen darstellen, sondern vielmehr Disziplinen, die sich gegenseitig informieren.

## Abstract (en)

Chronic pain poses a significant global socio-medical challenge causing significant costs. It is only since the mid-20th century that pain syndromes have been considered diseases in their own right. According to the definition of the International Association for the Study of Pain, pain is a complex, context-dependent—and hence modifiable—phenomenon. The philosophical view on pain is no less multi-facetted and allows for a wide range of viewpoints. This analysis aims at a characterisation of pain including a—mainly phenomenological and enactivist—philosophical perspective. The discourse will be guided by the concept of the limit(s) of the lived body: what is the relationship between pain and the perception of the lived body’s boundaries? Does a reciprocal influence exist? And may the perception be modified in order to reduce the patient’s suffering? These musings will also clarify that neurosciences and philosophy are not competing sciences, but rather inform each other.

## Einleitung

Chronische Schmerzsyndrome stellen eine große sozialmedizinische Herausforderung dar: In Deutschland war 2020 allein die Diagnose „Rückenschmerzen“ die zweithäufigste Ursache für krankheitsbedingte Fehltage [35]. Trotz stetigem Ausbau der schmerzspezifischen medizinischen Infrastruktur geht das Deutsche Institut für Medizinische Dokumentation und Informatik (DIMDI) „von einer Unterversorgung in der Größenordnung von rund 2500 Einrichtungen“ aus [9]. Würde eine solche Aufstockung der Versorgungsstruktur für chronische Schmerzpatienten das Problem lindern? Oder könnte sie es – z. B. im Sinne einer iatrogenen Fixierung – sogar verschlimmern? Vor dem Hintergrund dieser Komplexität und sektorenübergreifenden Problematik sollen die folgenden Überlegungen eine interdisziplinäre Herangehensweise an das Thema Schmerz darstellen, die die neurowissenschaftliche mit der philosophischen – phänomenologisch und enaktivistisch geprägten – Perspektive zusammenführt.

Auf der Grundlage der phänomenologisch geprägten Philosophie soll zunächst eine Charakterisierung des Schmerzes als grenzverletzendes Ereignis entworfen werden. Hierfür wird zunächst eine vorwiegend an Merleau-Pontys Auffassung von Körper und Leib angelehnte Theorie des leiblichen Subjekts und seiner körperlichen Grenzen – ihrer Ausdehnung, ihrer Verformbarkeit, ihrer Transparenz – entwickelt. „Grenze“ steht hier in sehr konventioneller (Sprachgebrauchs‑)Weise für die Linie zwischen dem räumlich-körperlichen Innen und Außen – und der Schmerz in Folge für das Widerfahrnis eines Zudringlichen, das als nicht mir zugehörig, als von außen kommend erfahren wird. Die Besonderheit des Schmerzes ist aber, dass er – im Gegensatz zu den meisten anderen Sinnesempfindungen –seinen Ursprung auch im Inneren des Körper haben kann, aber auch hier als fremd und grenzüberschreitend erlebt wird.

Unter Zuhilfenahme des Enaktivismus soll anschließend gezeigt werden, dass Schmerz, Körper und Kognition eine enge, sich wechselseitig beeinflussende Beziehung eingehen. Hierdurch kann dann abgeleitet werden, dass Schmerz und die Wahrnehmung der Ausdehnung und Beschaffenheit der leiblichen Grenzen in einem iterativen Wechselverhältnis stehen. Aus diesen Erkenntnissen sollen im letzten Teil des Artikels Vorschläge zur Integration philosophischer Modelle in die Schmerztherapie im Sinne des multimodalen Ansatzes abgeleitet werden.

## Schmerz als biomedizinisches und philosophisches Problem

Schon in der Definition der International Association for the Study of Pain (IASP) wird deutlich, dass Schmerz ein komplexes, kontextabhängiges, damit aber auch modifizierbares Phänomen darstellt: „An unpleasant sensory and emotional experience associated with, or resembling that associated with, actual or potential tissue damage“ [19]. Die Modifizierbarkeit des Schmerzes in Abhängigkeit von soziokulturellen Begleitumständen und affektiver Einfärbung ist eine auf neuroanatomischen und -physiologischen Voraussetzungen gut begründbare Entität. Schmerz ist nicht nur ein „von außen Gemachtes“ und passiv Weitergeleitetes wie in der mechanistischen Sichtweise Descartes (vgl. [8, S. 32–33]), sondern wird durch komplexe Regelkreise aktiv modifiziert. Die 1965 in ihrer ursprünglichen Form von Melzack und Wall beschriebene Gate-control-Theorie legt die Grundlage dieser neuronalen Schmerzmodulation dar [24]. Die Multimodalität des Schmerzempfindens spiegelt sich aber auch auf Ebene der kortikalen Schmerzmatrix wider. Im Zuge der Entstehung chronischer Schmerzen kommt es hier zu einer Restrukturierung mit verstärkter Aktivierung von Arealen, die an belohnungsorientiertem Lernen und emotionaler Verarbeitung beteiligt sind. Diese Assoziation kann in der Folge im Rahmen maladaptiver Lernprozesse zu einer zunehmenden Katastrophisierung mit Hilflosigkeit, Gedankenkreisen um den Schmerz und einer verstärkten Wahrnehmung desselben als bedrohlich führen [7]. Hier wird eine Dissoziation von Schmerz als neurophysiologisch verstandenem Prozess und Leiden im Sinne einer negativen Bewertung des Schmerzes evident.

Die philosophische Herangehensweise an den Schmerz ist mindestens ebenso komplex wie die biomedizinische und kann unter den verschiedensten Gesichtspunkten erfolgen. Welche Ebenen beinhaltet Schmerz? Ist er reine Perzeption? Impliziert er Bewusstsein oder gibt es unbewussten Schmerz (zum Beispiel im Schlaf)? Gehört der emotionale Affekt unbedingt zum Schmerz? Und muss dieser Affekt ein negativer sein? Beinhaltet Schmerz eine kognitive, wertende Komponente? Wie würde die Wertung ausfallen? Während es zu diesen Fragestellungen bereits sehr differenzierte philosophische Literatur gibt (für eine exzellente Zusammenstellung s. [5]), wurde der Interaktion von Schmerz und Leibesgrenze noch wenig Beachtung zuteil. Die folgenden Überlegungen sollen daher unter diesen Leitfragen erfolgen: In welcher Beziehung stehen Schmerz und die Wahrnehmung der Leibesgrenzen zueinander? Handelt es sich um eine wechselseitige Einflussnahme? Ist diese Wahrnehmung zugunsten des Schmerzpatienten modifizierbar?

Anhand dieser Überlegungen wird auch deutlich werden, dass die beiden Wissenschaften Neurobiologie und Philosophie mitnichten die Konkurrentinnen darstellen, als die sie zuweilen wahrgenommen werden, sondern vielmehr Disziplinen, die sich gegenseitig informieren. So hängt es wesentlich von den Komponenten ab, die wir der Definition von Schmerz für absolut zugehörig halten, welche neurobiologischen Bausteine wir in unser Model inkludieren: „A good theory of pain ought to capture both the phenomenological and biological features of pain and show how the latter illuminate the former“ [5]. Die unterschiedlichen Perspektiven und Methoden, derer sich diese Disziplinen bedienen – holistisch und systemorientiert im Bereich der Philosophie, unter Konstanthaltung der Rahmenbedingungen streng auf das Einzelfaktum gerichtet im Fall der Naturwissenschaften –, sollen sich im Bewusstsein ihrer jeweiligen Vorzüge und Nachteile gegenseitig ergänzen.

Im folgenden Teil dieser Arbeit soll unter Zuhilfenahme phänomenologischer Zugangsweisen herausgearbeitet werden, wie Schmerz auf das Individuum einwirkt und vice versa. Schönbächler definiert zwei Schnittstellen des Schmerzes: eine intrapersonale zwischen physischen und mentalen Aspekten des Schmerzes, und eine interpersonale zwischen Mensch und Umwelt [33]. Letztere – die Körper- bzw. Leibesgrenze – soll als Leitstruktur für die folgenden Überlegungen zum Schmerz dienen: Wie verändert er die leiblichen Grenzen in der Wahrnehmung des Betroffenen? Ist dieser Wirkvektor möglicherweise reziprok? Und könnte eine aktive Modifikation dieser Grenzwahrnehmung das Schmerzempfinden positiv oder negativ beeinflussen?

## Schmerz – eine phänomenologische Annäherung

Das Phänomen des Schmerzes ist für Überlegungen hinsichtlich der Abgrenzung des Körpers – bzw. des Leibes – bestens geeignet. Diese bildet die Grenze zur Umwelt, zu anderen Personen, sie definiert die Person, so wie sie von außen gesehen wird. Sie umhüllt die inneren Organe, bedeutet Schutz und Erfahrung der Unverletzbarkeit. Der Schmerz, der von außen kommt, stellt einen Angriff auf diese Grenze und das von ihr Implizierte dar. Der Schmerz, der von innen kommt, wird wie ein Eindringling, ein Fremdkörper wahrgenommen, der jedoch im Selbst generiert wird. Der Schmerz berührt daher die Grenze zwischen Innen und Außen von beiden Seiten. Und wie später gezeigt werden soll, ist diese Berührung nicht nur ein passives Tangieren, sondern ein aktives Gestalten mit wechselseitigem Einfluss: Schmerz bewirkt eine Verschiebung dieses Grenzbereichs, aber eine Modifikation der Grenze kann ihrerseits auch Schmerzen verstärken oder lindern.

Wie sollte nun diese Grenze zwischen dem Innen und dem Außen, die dem Körper doch unverrückbar gegeben zu sein scheint, verschiebbar sein? Um diese Frage zu beantworten, soll zunächst auf die Differenzierung zwischen Leib und Körper rekurriert werden, wie man sie in der phänomenologischen Tradition – insbesondere im Werk Merleau-Pontys – antrifft (s. v. a. [25]). In der phänomenologischen Auffassung ist das „wirkliche Subjekt des (chronischen) Schmerzes weder das körperlose Bewusstsein noch der physiologische Körper, sondern die verkörperte Subjektivität“ (s. [2, S. 180]). Diese verkörperte Subjektivität als Drittes, welches den cartesianischen Geist-Körper-Dualismus aufzuheben scheint, ist bei Merleau-Ponty der Leib (im französischen Original „le corps propre“).

Der Körper hingegen hat eine sehr starke biologische Konnotation. Er ist die sicht- und fühlbare Präsenz des Individuums in seiner Umwelt, er ist in seinen Ausmaßen quantifizierbar, in seinen inneren Vorgängen messbar. Und er ist vor allem eines: abgegrenzt. Die Isolation von Körper und Umwelt einerseits, aber auch von Geist und Körper andererseits überwindet Merleau-Ponty mit seiner Theorie des Leibes. Dieser ist die Juxtaposition zur biologisch geprägten Sichtweise auf den Körper. Er verwebt das Individuum mit seiner Umwelt – der dinglichen wie der interpersonellen – und überwindet die Dichotomie von Psyche und Physis, von Materie und Geist, von Innen und Außen. In dieser Sichtweise kommt dem Leib – im Gegensatz zur abgrenzenden Funktion des Körpers – eine verbindende Rolle zu, er ist das Medium der Vermittlung zwischen Kognition, Körper und Welt (vgl. [31, S. 53–54]).

Welches nun sind die Charakteristika des Leibes bei Merleau-Ponty? Der Leib nimmt eine Sonderstellung unter den Dingen ein. Er ist eine irreduzible Gegebenheit, durch die das Subjekt sowohl sich selbst als auch seine Umwelt wahrnimmt (vgl. [30, S. 241]). Neben der Rolle als wahrnehmendes, entdeckendes, fühlendes Agens gehört der Leib als Körper gleichzeitig zu den Objekten, welche die Umwelt ausmachen und somit wahrgenommen, berührt und erforscht werden können. Ihm wohnt somit einerseits etwas Gegenständliches inne – er öffnet sich dem forschenden Blick und explorativen Ertasten des Subjekts, so wie er für den Naturwissenschaftler als distinkte Entität existiert, die anatomisch beschrieben und physiologischen und biochemischen Experimenten unterzogen werden kann. Auf der anderen Seite ist der Leib eben auch *meiner*, mein Sein in der Welt, meine Möglichkeit, mit meiner Umgebung zu interagieren und diese wahrzunehmen. In diesem Sinne ist der Leib-Körper Verbindungsstück zwischen mir und meiner Welt – es ist in diesem Sinne, in dem ihn Merleau-Ponty als „Leib“ bezeichnet.

Aus dem Gesagten wird auch deutlich, dass die Grenzen des Leibes nicht so klar umrissen sein können wie die des Körpers. Es handelt sich hierbei eben um keine hermetisch abgeschlossene Einheit, sondern um eine Entität, die sowohl nach innen als auch nach außen ein Kontinuum formt und somit einen Konnex bildet: „Auf der anderen Seite jedoch bin ich nicht mein Körper, ich bin auch etwas anderes als mein Körper; denn er ist mir auch eine Gegebenheit, auf die ich mich in gegenständlicher Weise beziehen kann. Genau in diesem Sinne gehört mein Leib zu meiner Welt; … Mein Leib bildet so ein Kontinuum mit der mir gegenständlichen Welt, ohne dass er mir allerdings dadurch fremd würde. Mein Leib – das bin ich, und mein Leib gehört zu meiner Welt, die ich nicht bin, sondern zu der ich gehöre“ (s. [31, S. 55–56]).

Es ist also der Leib, der die Vermittlung des Subjekts mit der Welt unternimmt. In seiner Zwitterrolle zwischen Subjekt und Objekt *ist *der Leib auf der einen Seite in der Welt, auf der anderen *verhält* er sich aber auch zu ihr. Dies bedeutet auch, dass das Leibkonzept die intrasubjektiven Grenzen zwischen Wahrnehmen und Handeln bzw. zwischen Sensorik und Motorik verwischt: „In Merleau-Pontys Worten muss das In-der-Welt-Sein des Subjekts mit seinem Zur-Welt-Sein einhergehen. Darum muss das Subjekt bei Merleau-Ponty ein leibliches sein, denn nur der Leib vermag jene Doppelauflage zu erfüllen: der Leib ist sowohl *in* der Welt als auch *zur* Welt, …“ ([1]; kursiv JW). Der Leib nimmt diese Vermittlereigenschaft mittels eines untrennbar miteinander verwobenen Geflechts aus Wahrnehmung und Motorik im Sinne einer intentionalen Hinbewegung zu dieser Welt wahr: „Der Leib ist das Vehikel des Zur-Welt-seins, und einen Leib haben heißt für den Lebenden, sich einem bestimmten Milieu zugesellen, sich mit bestimmten Vorhaben identifizieren und darin beständig zu engagieren“ (vgl. [25, S. 106]).

Die Annahme einer untrennbaren Trias aus Kognition, Körper und Umwelt stellt die Basis einer weiteren philosophischen Strömung dar, welche erst in den vergangenen 30 Jahren aufgekommen ist. Wechselnd benannt als Verkörperung (oft mit dem englischen Terminus „embodiment“) oder Enaktivismus (der Begriff geht auf Maturana & Varela zurück, s. [23]) vereint sie phänomenologische und biomedizinische Ansätze, um zu einer Theorie des Leib-Körpers und seiner Interaktion mit dem Geist („mind“) einerseits und seiner Umwelt andererseits zu gelangen (für eine ausgezeichnete Übersicht zu diesem Thema s. [11, S. 9–102]). Es ist in dieser Interaktion, in der Kognition entsteht und stattfindet. Diese multiperspektivische Herangehensweise macht den Enaktivismus zur Untersuchung des Phänomens des Schmerzes besonders attraktiv: „The body, the world around it …, and the mind are a pain-making whole“ (s. [13, S. 103]). Die klassische Juxtaposition von Subjekt (Geist) und Objekt (Umwelt) mit einer von der jeweiligen Denkschule abhängigen Zuordnung des Körpers zu einer der beiden Seiten wird somit zugunsten einer intrinsischen Verbundenheit dieser Entitäten aufgegeben. Die daraus resultierende „kognitive Pointe der Philosophie der Verkörperung“ (vgl. [11, S. 16]) besteht darin, dass sowohl körperliche Fähigkeiten als auch die Umwelt eine modifizierende Wirkung auf die Kognition haben: „In its prenoetic role the body functions to make perception possible and to constrain intentional consciousness in various ways. … The body and its natural environment work together to deliver an already formed meaning to consciousness. … These conditions are both constraining and enabling factors produced in the ecological interaction between body and environment, …“ (s. [16], vgl. S. 138–141). Dies impliziert eine Abkehr von der traditionellen Auffassung, dass der Geist unidirektional den Körper und mittels dieses Körpers die Umwelt beeinflusst. Vielmehr besteht das Verständnis des Enaktivismus von Kognition gerade darin, „dass der Organismus sich die Welt aus der Perspektive seiner Identität erschließt und damit Bedeutung aktiv hervorbringt“ (vgl. [11, S. 85]). Somit gelangt der Enaktivismus schlussendlich zu einer zirkulären Konstitution von Organismus und Umwelt: Die Gestaltung der Umwelt erfolgt aktiv durch den Organismus vor dem Hintergrund seiner ökologischen Nische. Die Beschaffenheit dieser Nische wiederum wirkt auf den Organismus zurück. Es besteht eine „strukturelle Koppelung“ zwischen beiden (s. [11, S. 85]). Diese Auffassung legt nahe, dass Schmerz – als Umweltfaktor interpretiert –, Körper und Kognition untrennbar miteinander verwoben sind und in einer zirkulären Wechselwirkung stehen.

## Schmerz und Leibesgrenze: eine wechselseitige Abhängigkeit

Während die Körpergrenze also eine fixe Entität darstellt, welche für den eigenen Körper zumindest in Teilen visuell, vor allem aber sensibel über Berührung – sei sie taktil, thermisch oder nozizeptiv – wahrnehmbar ist, ist die Grenze des Leibes weniger eindeutig definiert. Vielmehr bildet sie einen Konnex mit der Umwelt des Subjekts und ist als solcher im Hineinhandeln in diese, in der Bezugnahme oder Intentionalität, wandelbar. Damit ist die Grundbedingung für die Annahme einer (gegenseitigen) Einflussnahme von Schmerz und Leibesgrenze gegeben.

Nachdem anhand der Theorien der Phänomenologie und des Enaktivismus gezeigt wurde, dass die wahrgenommene Begrenzung des Leibes in der sensomotorischen Interaktion von Subjekt und Umwelt modifizierbar ist, soll diese These nun hinsichtlich ihrer Bedeutung für das Schmerzerleben untersucht werden. Die folgende Abhandlung geht dabei von der Grundthese aus, dass der Leib als im Sinne des Enaktivismus „verkörperte Subjektivität“ (vgl. [2, S. 180], Anmerkung 1) das eigentliche Subjekt des Schmerzes darstellt. So wie es keinen Leib ohne Schmerz gibt, so ist auch ein Schmerz ohne Leib nicht vorstellbar: „It seems impossible to describe, or even imagine, a pain without reference to the body: disembodied pain does not make sense. But it is harder to say in what, precisely, the embodiment of pain consists. All sensations are trivially linked to the body in the sense that the originating sensory receptors lie within the body; but in the case of pain, the object of the sensation appears to be the body itself“ (Anmerkung: Die sprachliche Unterscheidung zwischen „Leib“ und „Körper“ kennt das Englische nicht, wobei für den „Leib“ gelegentlich der Begriff „lived body“ verwendet wird. Wo dies nicht der Fall ist, muss die exakte Bedeutung aus dem Kontext abgeleitet werden) [17].

Auch die neurowissenschaftliche Perspektive legt einen engen Zusammenhang von räumlicher und leiblicher Wahrnehmung, Motorik und Schmerzperzeption nahe: „… the perceptual quality of pain … [is] … profoundly linked to the spatial structure of the body. Thus, representations of the body and peripersonal space are important not only for motor responses to pain, but also for functional sensory organisation of pain itself“ [17]. Durch Kombination taktiler Stimuli auf der Hautoberfläche mit propriozeptiven Reizen, die die Stellung des Körpers im Raum codieren, findet eine Transformation von somatotopen in spatiotope Koordinaten statt. Hierdurch wird die Lokalisation von Reizen im Raum und eine gerichtete motorische Reaktion ermöglicht.

Im Folgenden soll daher die Hypothese spezifiziert werden, dass Schmerz die leiblichen Grenzen modifizieren kann. Zum anderen soll aber auch die Frage gestellt werden, ob eine Alteration dieser Grenzen Schmerz zu erzeugen oder zu lindern vermag. Sollte dies der Fall sein, könnten daraus therapeutische Konsequenzen erwachsen: Eine bewusste Manipulation der Leibesgrenzen müsste das Schmerzempfinden beeinflussen können. Aber auf welche Art und Weise alteriert der Schmerz die Leibesgrenzen? Erweitert er sie, lässt er sie schrumpfen oder verzerrt er sie? Für jede dieser Annahmen finden sich Argumente, die im Folgenden diskutiert werden sollen.

### Schmerz distendiert die Leibesgrenze

Der Schmerz lässt die betroffene Körperstelle hervortreten, er lenkt nahezu die gesamte Aufmerksamkeit auf sie. Sie sticht hervor, tritt aus der Transparenz des gesunden Leibes heraus: „Schmerz ist wehrloses Zurückgeworfensein auf den eigenen Körper, so zwar, dass kein Verhältnis mehr zu ihm gefunden wird. Die schmerzende Region scheint übergroß ausgebreitet und die übrigen Regionen zu überlagern und gänzlich zu verdrängen. … Brennend, bohrend, schneidend, stechend, klopfend, ziehend, wühlend, flimmernd wirkt der Schmerz als Einbruch, Zerstörung, Desorientierung, als eine in bodenlose Tiefe einstrudelnde Gewalt“ (s. [30, S. 352]).

Dieses Empfinden kann auch empirisch erhoben werden. In phänomenologisch orientierten „elicitation interviews“ mit Fibromyalgiepatienten finden sich Aussagen dahingehend, dass der betroffene Körperteil oder auch der ganze Körper als vergrößert wahrgenommen werden. Interessanterweise verläuft die Wahrnehmung des „near-space“ (peripersonaler Raum, gewöhnlich definiert als der Raum, der sich in Armreichweite befindet) konträr. Dieser wird als konstringiert wahrgenommen, zum Teil mit dem oppressiven Gefühl der Bedrängung und der Atemlosigkeit [36].

Umgekehrt führt die lokale Anästhesie eines Körperteils mitnichten dazu, dass dieser Teil als nicht mehr vorhanden wahrgenommen wird. Vielmehr wird er als verzerrt, eher vergrößert empfunden – ein Phänomen, welches die meisten Menschen bereits im Rahmen eines Zahnarztbesuches festgestellt haben dürften. Eine mögliche neurowissenschaftliche Erklärung für dieses Phänomen ist der Wegfall von tierexperimentell beschriebenen tonischen inhibitorischen Impulsen peripherer, schmerzleitender C‑Fasern auf den primären somatosensorischen Kortex mit dem Resultat temporärer größerer Überlappung benachbarter Areale kortikaler somatosensorischer Repräsentation und der Illusion der Vergrößerung eines Körperteils. Dies könnte zu einem Gefühl der Schwellung der betroffenen Gliedmaße führen [17, 29]. An dieser Stelle zeigt sich auch der Mehrwert einer Zusammenführung phänomenologischer und naturwissenschaftlicher Betrachtungen: die subjektive Wahrnehmungsbeschreibung leitet die Interpretation vorhandener naturwissenschaftlicher, experimenteller Daten.

### Schmerz verengt die Leibesgrenze

Diese These scheint vor allem auf den von außen kommenden Schmerz zuzutreffen. Stellen wir uns die Situation vor, in der uns ein Schlag trifft oder ein medizinischer Eingriff vorgenommen werden soll, bevor die lokale Betäubung ihre komplette Wirkung entfaltet hat. Die Reaktion ist Flucht – und wenn es keine Flucht im eigentlichen Sinne, aus der Situation heraus, geben kann, dann ist es eine Flucht nach innen. Der Körper kann seine Ausmaße nicht ändern, sodass es der Leib ist, der sich in sich selbst zurückzieht, weg vom externen Aggressor, auf dass die Angriffsfläche so klein wie möglich werde. Im Gegensatz dazu erscheint die Kontaktfläche mit der Welt zu nah, zu aufdringlich, zu penetrant, eine Intrusion der äußeren Welt in das Selbst (vgl. [32, S. 4]). Dietrich beschreibt den Schmerz als Störung einer Ordnung – so auch der Ordnung des Raums – im Sinne einer Erfahrung des Getroffenseins mit darauffolgender zentripetaler Rückzugsbewegung bzw. Abwendung von der Welt. Es besteht eine Unangemessenheit im Kontakt zwischen mir und der Welt: ein Zu-nah, Zu-heftig, Zu-viel (vgl. [2, S. 364–366]). Dieser Effekt ist nicht nur temporär, sondern projiziert über das Schmerzgedächtnis in die Zukunft: „Im Unterschied zu anderen Emotionen stumpft Schmerz nicht ab, im Gegenteil – der Leib sensibilisiert sich und versucht sich daher zu schützen, sei es durch vorwegnehmende Anspannung, Einkrümmung, Rigidität, durch Schonhaltungen, Rückzug oder implizite Vermeidung gefährlicher Situationen. All dies betont die Abgrenzung gegenüber der Umwelt. Der Leib entwickelt ein Gedächtnis seiner Verletzbarkeit und damit seiner Grenzen“ (s. [14, S. 321]).

Doch nicht nur die Grenzen des Leibes schrumpfen. Auch der auf die Objektwelt projizierte metrische Horizont wandelt sich. So beschreibt Carel am Leitsymptom der Dyspnoe, an der sie aufgrund einer chronisch progredienten Lungenerkrankung – der Lymphangioleiomyomatose – leidet, wie sich ihre Welt zurückzieht, ihr Bewegungsradius zunehmend kleiner und somit die Notwendigkeit, eigene Ziele anzupassen, immer größer wird: „The loss of freedom to act is experienced as the terrifying shrinking of one’s world. … this replacement world is anchored in a painful sense of wanting to inhabit another world, wanting another life, another body“ (s. [4, S. 118]). Dies lässt sich ohne Weiteres auf den Schmerz übertragen: Ein Rückenschmerz beispielsweise, der jeden Schritt zur Überwindung werden lässt, wird die in der Welt zurückzulegenden Distanzen ebenso um ein Vielfaches länger erscheinen lassen wie eine schwere Dyspnoe.

Durch Krankheit wird die Welt kleiner, unfreier, verschlossener. Dies limitiert einerseits die physische Bewegung. Im Sinne verkörperter Kognition verwehrt sich Carel aber auch gegen eine dualistisch geprägte Ansicht, dass der Geist unabhängig vom Körper funktionieren bzw. im Sinne einer Kompensation den eingeschränkten Körper sogar übertreffen könne: „Our embodiment determines our possibilities and delineates with extreme clarity that one is and is not permitted to do and be. The spirit is tethered to the body and its limitations cut deep into spiritual life“ (s. [4, S. 110]). Vielmehr beeinträchtigt eine Limitation des motorischen Schenkels der auf einem sensomotorischen Zirkel fußenden Kognition eben auch diese in ihrer Gesamtheit: „… there are instances, such as extreme or chronic pain, in which it is not possible to enjoy one’s freedom or imagination. In those cases one’s ability *to be* is indeed radically and inconsolably curtailed“ (s. [4, S. 84]; kursiv JW).

Der Schmerz beschränkt mit der Handlungsfreiheit, mit der Möglichkeit, „Selbstgestalter der Welt“ (s. [2, S. 171]) zu sein, somit in letzter Instanz auch die Optionen, Entscheidungen zu treffen und zu realisieren. In Rückgriff auf Heidegger limitieren Krankheit und Schmerz daher den Lebensvollzug des Menschen, dessen existenzielle Möglichkeiten: „Das Sein selbst, zu dem das Dasein sich so oder so verhalten kann und immer irgendwie verhält, nennen wir Existenz. … Die Existenz wird in der Weise des Ergreifens oder Versäumens nur vom jeweiligen Dasein selbst entschieden“ (s. [18, S. 12]). Die Möglichkeit, Entscheidungen zu treffen und Handlungen zu setzen, unterscheidet die menschliche Existenz vom reinen Vorhandensein, sie macht sein „Seinkönnen“ aus. Eine Limitation dieser Möglichkeit bedeutet unausweichlich eine existenzielle Beeinträchtigung.

### Wechselwirkung zwischen Grenze und Schmerz

Im vorherigen Abschnitt wurden zwei gegenläufige Entwicklungen skizziert: Zum einen soll Schmerz Leibesgrenzen erweitern, gleichzeitig jedoch verengen. Partiell lässt sich dieser Widerspruch abmildern, indem unterschiedliche Räume – der leibliche, der sich in Armreichweite befindliche peripersonale und die erweiterte Umwelt – betrachtet werden. So könnte man postulieren, dass sich die wahrgenommene Verengung vor allem auf den peripersonalen Raum sowie die erweiterte Umwelt statt auf die eigentlichen leiblichen Konturen bezieht, die Expansion dagegen exklusiv auf die Leibesgrenzen. Eine gänzliche Auflösung dieses Widerspruchs gelingt jedoch nicht, sodass die spezifische Ausformung der leiblichen Reaktion auf Schmerz vermutlich – auch – auf die jeweilige Situation und individuelle Disposition des Individuums zurückgeführt werden muss. Die vorherigen Ausführungen legen jedoch nahe, dass prinzipiell die Modulation leiblicher Grenzen durch Schmerz möglich ist. Dies führt zu der Frage, inwieweit eine aktive Modifikation der Grenzen von Raum und Leib Schmerz – ggf. auch therapeutisch – beeinflussen kann.

An dieser Stelle soll noch einmal betont werden, dass Schmerz keine reine Sensation ist, die den Verstand im Sinne eines streng linearen und unidirektionalen Leib-Seele-Modells passiv affiziert, sondern eine Wahrnehmung qua sinnhaftem Erfassen äußerer Eindrücke oder von Leibesempfindungen, welche durch zirkuläre sensomotorische Prozesse interpretiert und modifiziert wird. Wahrnehmung ist kein passiver Prozess, sie ist keine reine Repräsentation äußerer oder auch leiblicher Zustände im Gehirn des Individuums, auf die lediglich reagiert wird. Vielmehr ist Wahrnehmung ein aktiv-kreativer Prozess. Aufgrund dieser Zusammenhänge ziehen Veränderungen des Körpers auch Veränderung im Sich-seiner-selbst-Bewusstsein und im Sein-in-der-Welt nach sich (s. [4, S. 27]). Dies trifft – wie oben dargestellt – auch für die Modulation der Leibesgrenze durch Schmerz zu. Aber der Wahrnehmungsvektor zwischen Leibesgrenzen und Schmerz kann in beide Richtungen weisen. So wie Schmerz diese Grenzen zu verändern oder aufzulösen vermag, kann eine Verzerrung bzw. Auflösung derselben den Schmerz begünstigen oder gar generieren (s. [17, S. 10]).

Diese Hypothese wird u. a. durch neurowissenschaftliche experimentelle Daten gestützt, die die visuelle Analgesie betreffen. Letztere beschreibt den Effekt, dass allein die Beobachtung eines schmerzenden Körperteils den Schmerz lindern kann. Es konnte gezeigt werden, dass eine Grenzexpansion durch eine scheinbare, durch Spiegel induzierte Vergrößerung der eigenen Hand den Effekt der visuellen Analgesie verstärkt, während eine Verkleinerung ihn mindert [17, 22].

Zum anderen tragen Beobachtungen an chronischen Schmerzpatienten zu der Überlegung bei, inwiefern das Grenzerleben des Leibes schmerzmodifizierend wirken kann. Viele dieser empirischen Daten wurden an Patienten gewonnen, die an einem „chronic regional pain syndrome“ (CRPS) leiden. Eine CRPS-Patientin beschrieb ein Fremdheitsgefühl und eine Alteration des Lagesinns in einem gebrochenen Arm *vor* Einsetzen des klassischen CRPS-Schmerzes. Zwar beweist diese Beobachtung noch keine Kausalität hinsichtlich einer Schmerzinduktion durch Verzerrung des Körperempfindens. Andererseits konnte auch für das CRPS vor dem Hintergrund der visuellen Analgesie eine Modulation des Schmerzempfindens durch eine mittels Spiegelung induzierte Illusion gezeigt werden: Eine visuelle Vergrößerung der betroffenen Extremität verstärkte den Schmerz, eine Verkleinerung linderte ihn [3, 17]. Diese Beobachtung stützt die Annahme einer wechselseitigen Interaktion von Schmerz und leiblichem Grenzempfinden.

Vor diesem Hintergrund sind solche Daten interessant, die nahelegen, dass eine Reorganisation bzw. Korrektur des Körperschemas schmerzlindernd wirkt. Bei Patienten mit Phantomschmerz konnte gezeigt werden, dass durch ein sensorisches Trainingsprogramm, bei dem die Patienten Ort und Frequenz von auf dem Stumpf aufgebrachten elektrischen Stimuli unterscheiden sollten, sowohl subjektives Schmerzempfinden gelindert als auch gemessene kortikale Reorganisation induziert werden konnte – und zwar effektiver als durch konventionelle medikamentöse Maßnahmen [12]. Gezieltes Training der Diskrimination taktiler Reize wirkte schmerzmindernd, möglicherweise über eine Normalisierung pathologischer Reorganisation [27]. Hier zeigen sich mögliche therapeutische Angriffspunkte auf das Schmerzempfinden durch Stärkung des Körperschemas mittels sensorischen Trainings auf.

Dieser aktiven Beeinflussbarkeit des Schmerzes nähert sich Olivier von einer phänomenologischen Warte. Er wählt den Ansatz des „embodiments“, wenn er sagt: „perception is no epiphenomenon, but a bodily capacity. A change of perception means a change of our bodily state“ (s. [28, S. 188]). Wahrnehmung ist also ganz und gar an den Leib gebunden, er ist der Mittler zwischen Innen und Außen. Beide – fungierender Leib und Wahrnehmung – wirken wechselseitig aufeinander ein. Schmerz reduziert den Raum und löst ihn auf (vgl. [28, S. 134]). Die Konstriktion des peripersonalen Raums trägt zu dieser Reduktion bei, ebenso wie der Rückzug des Körpers vom Schmerz: „… the body takes a safeguarding position or flees into sheltering rooms and closed corridors …“ (s. [28, S. 138]). Dieses Entfliehen kann das leidende Subjekt in die Isolation und die Vereinsamung führen. Aber die Möglichkeit der Einflussnahme ist eben auch in der gegensätzlichen Richtung denkbar: Eine bewusste Modulation des In-die-Welt-Hineinhandelns vermag Schmerzen zu lindern. Olivier postuliert hier am Beispiel des französischen Schriftstellers Alphonse Daudet, der jahrelang stark unter den Folgen einer Syphilis-Erkrankung litt und diese in dem Buch „Im Land der Schmerzen“ festhielt, dass durch eine bewusste Verhaltensänderung gegenüber Raum und Ort auch die Art und Qualität des Schmerzes verändert werden kann: „Taking the perspective outward, the capturing onset of his pain changed. The pain lost something of its all-absorbing quality“ (s. [28, S. 138]).

## Schmerz und die leibliche Transparenz

Bisher wurden die leiblichen Grenzen unter dem Kriterium ihrer Ausdehnung betrachtet. Eine weitere Kategorie, die zur Charakterisierung herangezogen werden kann, ist die der Transparenz. Der Leib oszilliert zwischen zwei Polen: der Transparenz und der Ständigkeit. Während ich mich von umgebenden Gegenständen abwenden kann, ist der Leib nicht als abwesend denkbar: „Seine Ständigkeit ist keine solche der Welt, sondern Ständigkeit „meinerseits“. Diese Ständigkeit besagt, „Daß ich niemals ihn eigentlich vor mir habe, daß er … immer am Rand meiner Wahrnehmung bleibt und dergestalt mit mir ist.“ (s. [25, S. 115]). Auf der anderen Seite nehmen wir den Leib im Handeln nur marginal wahr, er ist transparent. Im gesunden Zustand ist unser Körper das Vehikel, das es uns ermöglicht, die Welt zu erleben, das „… Wunder der Selbstvergessenheit“ (s. [15, S. 126]). Hierbei bleibt er unsichtbar, das Erleben des Selbst erfolgt letztlich durch die Interaktion – Aktion und Reaktion – mit der Umwelt: „… bodily presence is of a highly paradoxical nature. While in one sense the body is the most abiding and inescapable presence in our lives, it is also essentially characterized by absence. That is, one’s own body is rarely the thematic object of experience“ (s. [20, S. 1]). Hier wird eine idealisierte Sicht auf den gesunden Körper vorausgesetzt. Auch im gesunden Zustand ist der Körper allenfalls selten transparent: Mal fühlen sich die Muskeln nach längerem Verharren in einer Position etwas steif ein, der Gürtel einer zu engen Hose drückt ein wenig, der Wind streift über Haare und Gesicht, die Sonne brennt auf der ihr zugewandten Körperstelle usw. In der Krankheit und im Schmerz wird der Körper allerdings in hohem Maße intransparent, er präsentiert sich uns im Sinne eines aufgezwungenen Bewusstseins: „… in addition to deliberate reflection on one’s own body, the body manifests itself in consciousness in certain ’limit-situations’ …“ (s. [16, S. 28–29]).

Thomas Fuchs unterscheidet in Anlehnung an Erich Fromm zwischen „Körper haben“ und „Leib sein“. Im Zustand des Leib-Seins ist der Körper transparent, selbstverständlich, nichtgegenständlich – er ist „die Bewegung des Lebens selbst“ und steht in ständiger Wechselbeziehung zu seiner objekthaften und sozialen Umwelt. Zum Körper jedoch „wird der Leib (…) vor allem in den Störungen des gewohnten Lebensvollzugs, …“, als „affizierbarer oder pathischer Leib“ (s. [34, S. 86]). Beispiele hierfür sind Hunger, Durst oder eben auch Schmerz – sie führen dazu, dass wir den Körper in seiner Stofflichkeit bewusst erleben – und uns selbst somit als biologische, verletzliche und sterbliche Kreaturen. Eine Störung der Transparenz des Körpers durch Krankheit oder Schmerz führt nicht nur zur Gewahrwerdung des Körpers. Vielmehr wird die Stelle der Dysfunktion Zentrum der Aufmerksamkeit, sie macht sich über die Maße bemerkbar und drängt sich in den Vordergrund (s. [4, S. 55–60]). Dies wird dadurch mitbedingt, dass der Schmerz eine der wenigen Wahrnehmungen ist, die auch bei längerer Persistenz nicht zu einer Habituierung führen. Diese phänomenologische Beobachtung wird durch die funktionelle Bildgebung untermauert: Ein frontoparietozinguläres Netzwerk, welches auf die Relevanz und Neuheit sensorischer Reize reagiert und somit mutmaßlich mit deren Salienz korreliert, zeigt bei nicht schmerzhaften Reizen lediglich eine passagere Antwort, bei schmerzhaften jedoch eine anhaltende [10].

Eine weitere Konsequenz dieses erzwungenen Rückzugs ist der Verlust der Selbstverständlichkeit der Leiblichkeit mit der Folge der Zerstörung des Vertrauens in das Selbst. Mit dem Verlust der Transparenz des Körpers geht ein Kontrollverlust einher. Statt mittels des Leibes in Kontakt mit der Welt treten zu können, muss sich das Subjekt der Verfügung durch den Körper unterwerfen: „… Das Einmannorchester der Schmerzen, das bin ich“ (s. [6, S. 42]). Wenn der Leib in Rückgriff auf Husserl die präreflektive Instanz des Selbst, der Nullpunkt der Orientierung, Willensorgan und Instrument freier Bewegung ist und somit das Sein definiert (s. [32, S. 151]), bedeutet sein Zurücktreten hinter den Körper im Rahmen schwerster Erschütterungen, starker Schmerzen zwangsläufig Desorientiertheit, Einschränkung des Willens und seiner Ausübung und letztlich Auflösung des Selbst-Seins. An die Stelle des Vertrauens in den Körper, des „bodily trust“, des „Ich kann“, rücken Zweifel – „bodily doubt“. Die Ausrichtung der Aufmerksamkeit ändert sich von einer zentrifugalen, auf die Umgebung gerichteten zu einer zentripetalen, introspektiven (s. [32, S. 175–176]).

In dem Maße, in dem der schmerzende Körper in den Vordergrund tritt und die Kapazitäten der Wahrnehmungen auf sich zieht, wird er zum Tyrannen, der die Verbindung mit dem Außen unterminiert: „Schmerz, du mußt mir alles sein. Laß mich in dir all die fernen Länder finden, die zu besuchen du mir nicht mehr erlauben wirst. Sei du meine Philosophie, meine Wissenschaft“ (s. [6, S. 57]). In dieser Gefangennahme durch den Schmerz, die den Körper in seiner ganzen stofflichen Opazität vor den Leib treten lässt, werden wir unserer Fragilität – auch im Sinne unserer Sterblichkeit – gewahr. Der Schmerz ist also immer auch ein Hinweis auf unseren eigenen Tod als Extremum der Verunsicherung: „Das größte Übel, das Schlimmste, was überall gedroht werden kann, ist der Tod, die größte Angst Todesangst“ (s. [21, S. 593]).

Diese Reifizierung von Körper und Schmerz kann unter Umständen durch rein organisch ausgerichtete diagnostische und therapeutische Maßnahmen verstärkt werden. Die Objektifizierung des Körpers und die Externalisierung, Verdinglichung des Schmerzes im Rahmen dieses Prozesses machen diesen zu einem vom Subjekt getrennten Ding, welches mit den Augen der Wissenschaft betrachtet und kontrolliert werden kann: „A change of perception means a change of the quality of our pain“ (s. [28, S. 166]). Dieser Prozess der Konversion von Schmerz in eine Diagnose spiegelt auch eine interessante medizinhistorische Entwicklung wider, die David Morris in seiner Abhandlung „The Culture of Pain“ darlegt: „Pain on this view [of Western medicine, JW] is a message composed, sent, and delivered by illness. … alongside this familiar view it [the medical revolution, JW] introduces a basic change in perspective from which we see that pain is sometimes completely illegible. … Now the message is the illness. … We might think of the transformation of pain from symptoms to diagnosis … as representing a kind of Copernican revolution within medicine.“ Für Morris geht diese Kopernikanische Wende Hand in Hand mit dem Perspektivwandel einher, dass es sich bei Schmerz um eine Perzeption, keine Sensation handelt [26]. Dieser Perspektivwechsel kann im Sinne einer Kontrollüberzeugung eine therapeutische Funktion haben: „Diagnostic labels are hard currency for people living with persistent pain“ (s. [28, S. 312]). Er kann jedoch auch eine weitere Entfremdung vom eigenen Leib nach sich ziehen, den Schmerz zu einer Entität, einem „Es“ werden lassen, das zu einem unabhängigen – und somit unkontrollierbaren – Handelnden mutiert.

Rysewyck fasst diese Aspekte zusammen und gibt ihnen eine positive Wendung. Er sieht chronischen Schmerz zunächst als depersonalisierendes Erlebnis, das die fundamentale Beziehung der Person zu ihrem Körper im Sinne der Dienstbarkeit des Körpers, zu ihrer Umgebung, zu anderen Personen und zu sich selbst zerrüttet. Allerdings könne sich auch ein repersonalisierendes Erlebnis einstellen, da es die Person zwingt, diese Beziehungen wiederherzustellen (s. [32, S. 149, 153–154]).

## Fazit für die Praxis

- Wie könnte solch ein repersonalisierendes Erlebnis aussehen bzw. wäre es durch gezielte therapeutische Intervention induzierbar?
- Sowohl aus biomedizinischer als auch phänomenologischer Perspektive besteht ein Zusammenhang zwischen dem Empfinden der leiblichen Grenzen und dem Schmerzerleben. So wie Schmerz die Wahrnehmung der Leibesgrenzen modifiziert, kann die Alteration der Leibesgrenze Schmerz induzieren bzw. lindern. Aus diesem wechselseitigen Zusammenhang lässt sich die These ableiten, dass die Schmerzintensität durch eine bewusste Reorganisation des Raums sowie durch „Neuerlernen“ der Leibesgrenzen positiv beeinflusst werden kann.
- Ermutigende Daten, wie diese kognitive Modulation im Sinne einer Erweiterung des Ausblicks auf die Welt über den Schmerz hinaus vonstattengehen könnte, liegen für die kognitive Verhaltenstherapie, das Achtsamkeitstraining und verwandte Praktiken wie Entspannungstechniken, Biofeedback und Meditation vor. Diese Ansätze sollten daher Teil der multimodalen Schmerztherapie sein.
- Die hier getätigten philosophischen Ausführungen sollen auch dazu dienen, das Konzept der multimodalen Schmerztherapie aus dieser Perspektive heraus zu untermauern. Diesbezüglich kann es auch für den therapeutischen Dialog hilfreich sein, wenn die oben dargestellten Mechanismen der Reifizierung des Schmerzes und des Leibes und ihre eventuelle Induktion durch undifferenzierte medizinische Maßnahmen (im Sinne einer rein organischen, technischen Medizin) zur Sprache kommen. Ebenso legt der phänomenologische Ansatz über das Leibgedächtnis Erklärungsmodelle hinsichtlich der emotionalen Einfärbung von Vergangenheit und Zukunft durch aktuelles Schmerzerleben nahe. Für die praktische Anwendung legt die gegenseitige Beeinflussung von Leibempfinden und Schmerz nahe, dass eine Stärkung des Körperschemas mittels sensorischen Trainings einen therapeutischen Angriffspunkt bieten kann, um Schmerzen zu lindern.
- Der phänomenologische Ansatz unterstützt das biopsychosoziale Denkmodell auch durch die Hervorhebung der Rolle der Sensomotorik. Über die Einschränkung der Motorik durch schmerzhafte sensorische Empfindungen wird die Welt kleiner, kognitive Freiheit und Handlungsautonomie werden eingeschränkt. Eine bewusste Modulation des In-die-Welt-Hineinhandelns vermag Schmerzen zu lindern. Das Ziel von Therapie ist es daher auch, verloren gegangene Autonomie wiederherzustellen.
- Wie oben geschildert evoziert Schmerz unsere Körperlichkeit im Sinne der Materialität – und somit unsere Vergänglichkeit. Ein erweitertes Grenzverständnis legt daher die körperliche Niederlage, den Tod nahe, der dem Schmerz als Memento mori unausweichlich innewohnt und einen Widerhall in der „Grenzsituation“ der existenziellen Philosophie, wie sie von Jaspers entwickelt und unter anderem von Frankl und Yalom aufgenommen wurde, findet. Eine Ausweitung des Grenzbegriffs über den in der Einleitung definierten hinaus führt daher über die existenzielle Philosophie bzw. Psychotherapie zu weiteren therapeutischen Ansätzen, die in die multimodale Schmerztherapie integriert werden könnten.
